# Maximal exercise and plasma cytochrome P450 and lipoxygenase mediators: a lipidomics study

**DOI:** 10.14814/phy2.14165

**Published:** 2019-07-15

**Authors:** Benjamin Gollasch, Inci Dogan, Michael Rothe, Maik Gollasch, Friedrich C. Luft

**Affiliations:** ^1^ Experimental and Clinical Research Center (ECRC) a Joint Institution between the Charité University Medicine Max Delbrück Center (MDC) for Molecular Medicine Berlin‐Buch Germany; ^2^ HELIOS Klinikum Berlin‐Buch Berlin Germany; ^3^ LIPIDOMIX GmbH Berlin Germany; ^4^ Max‐Delbrück Center (MDC) for Molecular Medicine in the Helmholtz Association Berlin Germany

**Keywords:** Eicosanoids, exercise, fatty acids, lipidomics

## Abstract

Epoxides derived from arachidonic acid (AA) are released during exercise and may contribute to vasodilation. However, exercise may also affect circulating levels of other epoxides derived from cytochromes P450 (CYP) monooxygenase and lipoxygenase (LOX) pathways, many of whose exhibit cardiovascular activity in vitro. The effects of exercise on their levels have not been documented. We tested the hypothesis that acute, maximal exercise would influence the plasma concentrations of these vasoactive substances. We measured plasma CYP and LOX mediators derived from both the *n* − 3 and *n* − 6 fatty acid (FA) classes in healthy volunteers before, during and after short‐term exhaustive exercise. Lipid mediators were profiled by means of LC–MS/MS tandem mass spectrometry. A maximal Bruce treadmill test was performed to voluntary exhaustion. Exhaustive exercise increased the circulating levels of epoxyoctadecenoic (12,13‐EpOME), dihydroxyeicosatrienoic (5,6‐DHET), dihydroxyeicosatetraenoic acids (5,6‐DiHETE, 17,18‐DiHETE), but had no effect on the majority of CYP and LOX metabolites. Although our calculations of diol/epoxide ratios revealed preferred hydrolysis of epoxyeicosatrienoic acids (EEQs) into their diols (DiHETEs), this hydrolysis was resistant to maximal exercise. Our study is the first documentation that bioactive endogenous *n* − 3 and *n* − 6 CYP lipid mediators are released by short‐term exhaustive exercise in humans. In particular, the CYP epoxy‐metabolite status, 12,13‐EpOME/DiHOME, 5,6‐EET/DHET, 5,6‐EEQ/DiHETE and 17,18‐EEQ/DiHETE may contribute to the cardiovascular response during maximal exercise.

## Introduction

**Figure 1:**
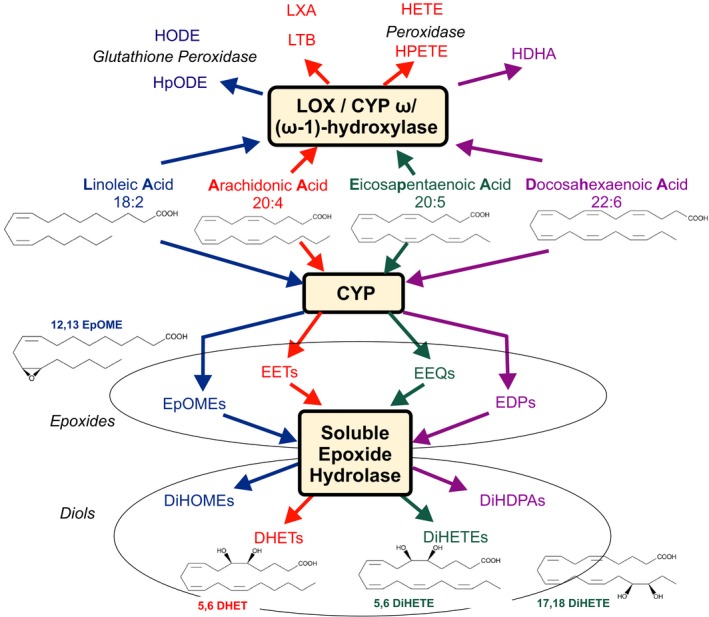
Cytochrome P450 epoxygenase (CYP) and 12‐ and 15‐lipoxygenase (LOX)/CYP (omega‐1)‐hydroxylase pathways. Linoleic (LA), arachidonic (AA), eicosapentaenoic (EPA) and docosahexaenoic acids (DHA) are converted into epoxyoctadecanoic acids (EpOMEs, e.g., 12,13‐EpOME), epoxyeicosatrienoic acid (EETs), epoxyeicosatrienoic acids (EEQs) and epoxydocosapentaenoic acids (EDPs) by CYP, respectively. EpOMEs, EETs, EEQs, and EDPs primary metabolic fate its conversion into the dihydroxyoctadecanoic acids (DiHOMEs), dihydroxyeicosatrienoic acids (DHETs, e.g. 5,6‐DHET), dihydroxyeicosatetraenoic acids (DiHETEs, e.g. 5,6‐DiHETE, 17,18‐DiHETE) and dihydroxydocosapentaenoic acids (DiHDPAs), respectively, by the soluble epoxide hydrolase (sEH) enzyme. LA, AA, EPA, and DHA are converted to hydroperoxylinoleic acids (HpODEs), hydroxyoctadecadienoic acids (HODEs), leukotriene B (LTB), lipoxin A (LXA), hydroxydocosahexaenoic acids (HDHAs), hydroperoxyeicosatetraenoic acids (HPETEs) and hydroxyeicosatetraenoic acids (HETEs) by LOX, CYP omega/(omega‐1)‐hydroxylase and peroxidase pathways. The metabolites measured within these pathways track the changes observed in LA, AA, EPA and DHA, respectively. Arrows demarcate metabolic pathways evaluated in response to short‐term maximal exercise.

Vasoactive substances, including prostaglandins, nitric oxide (NO), adenosine, adenosine 5′‐triphosphate (ATP), are released from contracting skeletal muscle and vascular endothelium and may contribute to vascular relaxation (Clifford and Hellsten 2004). However, other vasoactive substances may also be involved. In this regard, arachidonic acid (AA) can be converted into vasoactive metabolites *via* the cytochromes P450 (CYP) mono‐oxygenase and lipoxygenase (LOX) pathways (Fig. 1). In particular, the CYP pathway converts AA into four distinct epoxyeicosatrienoic acid (EET) regio‐isomers, namely 5,6‐, 8,9‐, 11,12‐ and 14,15 EET (epoxides). EETs can cause hyperpolarization of smooth muscle cells and relaxation by opening of Ca^2+^‐activated K^+^ (BK) channels (Zhu et al. 1995; Spector 2009). Activation of this pathway is partially or totally unresponsive to inhibitors of cyclo‐oxygenases that metabolize AA to 5‐, 12, and 15‐hydroxyeicosatetraenoic acid (HETE) (Fig. 1), prostaglandins, prostacyclin (PGI_2_), thromboxane and leukotrienes and inhibitors of NO synthase (Bauersachs et al. 2002; Busse et al. 2002). Based on these properties, EETs are considered candidates for endothelium‐derived hyperpolarizing factors (EDHFs) (Campbell et al. 1996), whose release is triggered by Ca^2+^‐induced activation of the CYP pathway (Graber et al. 1997) and shear stress (Campbell and Fleming 2010).

It has been suggested that EDHFs can contribute to increases in skeletal muscle blood flow that occur during exercise (Hillig et al. 2003). These properties could be essential to counteract the vasoconstrictor effects of concurrent increases in muscle sympathetic nerve activity and circulating vasoconstrictor substances during exercise. EETs are attractive mediator signals in this scenario (Campbell and Fleming 2010). In addition, dihydroxyeicosatrienoic acids (DHETs, diols), the stable metabolites of EETs produced by soluble epoxide hydrolase (Fig. 1), have been identified to cause vasodilation in some regional circulations, for example, the heart (Federation for Laboratory Animal Science Associations, XXXX). Beside AA, other *n* − 3 and *n* − 6 fatty acids (FA) are metabolized by CYP and LOX pathways to bioactive substances (Fig. 1). As such, linoleic acid (LA) is metabolized to epoxyoctadecanoic acids (EpOMEs) by CYP epoxygenases and further to dihydroxyoctadecanoic acids (DiHOMEs) by soluble epoxide hydrolase, which may play an important role in cardiac ischemic events (Bannehr et al. 2019). Eicosapentaenoic acid (EPA) is metabolized to epoxyeicosatetraenoic acids (EEQs), which can also cause hyperpolarization of vascular smooth muscle cells and relaxation by opening BK channels (Hercule et al. 2009). Other candidates are epoxides, and diols produced from the actions of lipoxygenases (LOXs), CYPs, and epoxide hydrolases using unsaturated FAs from other *n* − 3 [e.g., alpha‐linolenic (aLA)] and *n* − 6 [e.g., linoleic (LA), dihomo‐ alpha‐linolenic (dgLA), arachidonic (AA)] series as substrates (Fig. 1). Thus, increases in their concentrations during exercise may also contribute to corresponding increases in blood flow and cardiovascular response.

Although the effects of dietary EPA/docosahexaenoic acid (DHA) supplementation on plasma CYP‐eicosanoid profile have been reasonably well described (Fischer et al. 2014), reports of circulating levels of CYP metabolites in response to exercise in humans are rare and have been focused only on AA metabolites (i.e., EETs and DHETs) (Giordano et al. 2011). Giordano et al. (2011) found that submaximal exercise testing on a bicycle ergometer caused an increase in plasma levels of 8,9‐DHET, 11,12‐DHET and 14,15‐DHET. However, the effects of short‐term maximal exercise on a wide array of *n* − 6 and *n* − 3 metabolites remain unreported to the best of our knowledge. In this study, we tested the hypothesis that exhaustive exercise would influence plasma concentrations of epoxides derived from cytochromes P450 (CYP) monooxygenase and lipoxygenase (LOX) pathways.

## Methods

Prior to participation in the study six healthy volunteers (5 male and 1 female; age 38 ± 15 years; body mass index 27.9 ± 6.6 kg/m^2^) signed informed consent forms which outlined the procedures to be taken and the possible risks involved (Gollasch et al. 2019). The study was approved by the Charité University Medicine institutional review board on the use of humans in research. All subjects were nontrained. They were not taking medications. Recruitment was primarily via person*‐*to‐person interview. Following a routine physical examination at baseline levels each subject underwent a maximal treadmill Bruce test according to guidelines of the German Society of Cardiology (Bruce et al. 1973; Trappe and Lollgen 2000). The test was preceded by 2 × 3 min warm up periods (stages 1 and 2 of the Bruce protocol), during which treadmill speed was maintained at a constant speed of 2.7 km/h and at zero or 5% grade as shown in Table S1. Treadmill speed and grade were then increased at 3‐min intervals. The test was terminated when the subjects informed the investigator that they could no longer proceed. Workload was assessed in metabolic equivalents (METs) as shown in Table S1.

**Figure 2:**
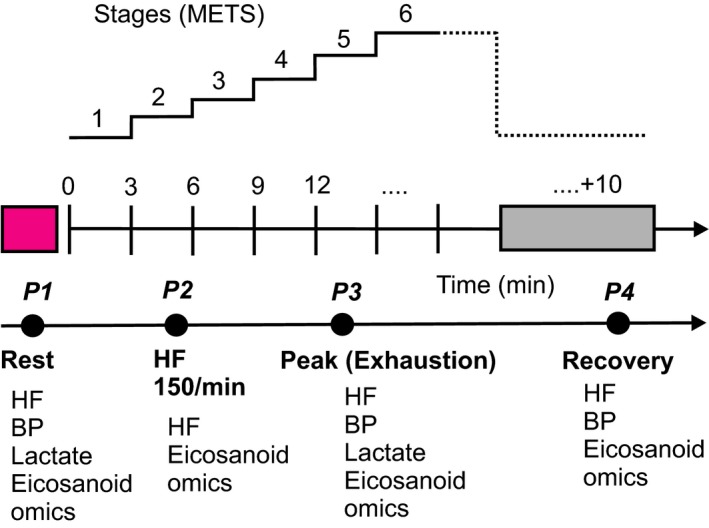
Schematic illustration of the standard Bruce protocol used. HF, heart rate; BP, blood pressure; eicosanoid fatty acids; METs, metabolic equivalents of task. *P1* (rest), *P2*,* P3* (peak, exhaustion), *P4* (recovery); time points used to measure HF, BP, eicosanoid lipid profiles, lactate.

Heart rates were monitored continuously by the heart‐rate monitor worn around the subject's torso (Polar T31, Polar Electro, Kempele, Finland) throughout the tests. Arterial pressure was measured in each subject while sitting prior to the exercise test (−10 min), after termination of the test (exhaustion), and 10 min recovery after the end of the running test *via* a sphygmomanometer (Critikon, Inc., Johnson & Johnson, New Jersey, USA), which comprised an inflatable (Riva‐Rocci) cuff placed around the upper arm. Venous blood was collected from a catheter placed in a contralateral forearm vein (i.e., the antecubital vein) of each subject in the sitting position prior to the exercise test (−10 min), after termination of the test (exhaustion), and 10 min recovery after the end of the running test (Fig. 2). An additional blood sample was collected in each subject during running when the heart rate reached 150 beats per minute. We did not measure blood pressure at this time point (HF 150) because valid blood pressure measurements could not be obtained during running using the above sphygmomanometer. All samples were analyzed for plasma eicosanoids. Red blood cells (RBCs) were separated from EDTA blood by centrifugation and eicosanoids in plasma were determined by liquid chromatography mass (HPLC‐MS) spectrometry described in (Fischer et al. 2014). Serum lactate was determined in blood samples obtained from ear lobe at rest and at maximal workload.

We performed sample size calculation for a difference in means in DHETs (Giordano et al. 2011). We found that our study would require a sample size of 6 (number of pairs) to achieve a power of 80% and a level of significance of 5% (two sided), for detecting a mean of the differences of 0.15 between pairs (8,9‐DHET), assuming the standard deviation of the differences to be 0.09 (Giordano et al. 2011).

Descriptive statistics were calculated and variables were examined for meeting assumptions of normal distribution without skewness and kurtosis. In order to determine statistical significance between the trials at the various time intervals, one‐way repeated measures analysis of variance (ANOVA) was conducted and the 0.05 level of significance (*P*) was chosen. The analysis included Mauchly's test of sphericity followed by applying the test of within subjects effects with Greenhouse–Geisser correction to ensure sphericity assumption. When significant differences were found, Tukey's honestly significant difference post hoc test was used for pairwise comparisons. Planned hypotheses (one‐tailed or two‐tailed paired *t*‐tests as appropriate) were tested to follow up the initial ANOVA findings.

All data are presented as mean ± SD. All statistical analyses were performed using SPSS Statistics software (IBM Corporation, Armonk, NY) or All‐Therapy statistics beta (AICBT Ltd, Vancouver, Canada).

## Results

### Hemodynamics

**Figure 3:**
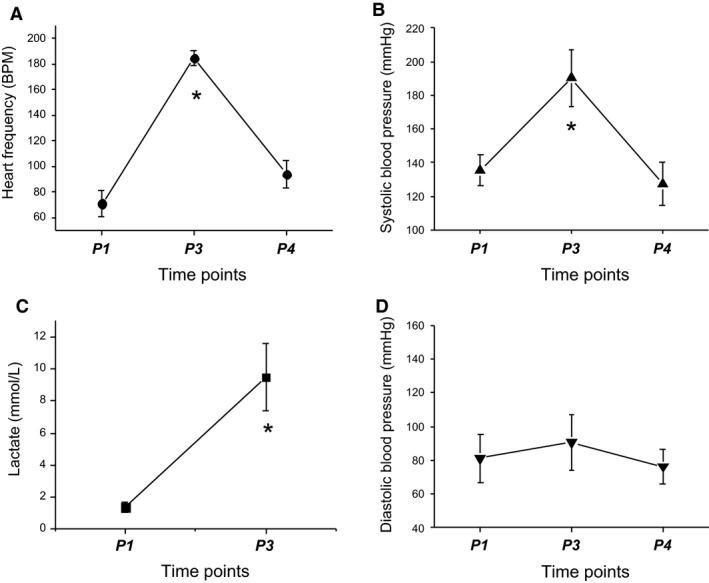
Effects of short‐term exhaustive exercise on hemodynamics. Means ± SD at time points *P1* (rest), *P3* (exhaustion), *P4* (recovery).

Figure 3 shows the results on the effects of acute exercise on heart frequency and blood pressure. The values include measurements prior to the exercise test (−10 min, *P1*), after termination of the test (exhaustion, *P3*), and 10 min after the end of the running test (recovery period, *P4*). An additional blood sample was collected in each subject during running when the heart frequency reached 150 beats per minute (*P2*) (Gollasch et al. 2019). At maximal workload, heart rate, and systolic blood pressure increased from 71 ± 10 (baseline) to 185 ± 6 beats per minute and from 135.3 ± 9.1 to 190.3 ± 16.6 mmHg, respectively (*P* < 0.0001 each, *t*‐tests). Diastolic blood pressure did not change (81.2 ± 14.4 vs. 90.7 ± 16.4 mmHg, *P* > 0.05). Maximal workload occurred at 13.50 ± 1.97 METs (*P3*) in the individuals. The corresponding Bruce stage was 6.33 ± 0.82. Lactate levels increased from 1.38 ± 0.30 mmol/L at rest (*P1*) to 9.49 ± 2.10 mmol/L at exhaustion (*P3*) (*P* < 0.0001, *t*‐test), which is consistent with a robust metabolic response leading to significant lactate acidosis (Gollasch et al. 2019).

### CYP and LOX mediators

Since the impact of acute exercise on circulating *n* − 3 and *n* − 6 CYP and LOX‐mediator levels is unknown, we used an explorative statistical approach on measured free CYP‐ and LOX‐dependent metabolites. We first measured the individual‐free metabolites in plasma at different time points: rest (baseline), the time when heart rate reached 150 beats per min, exhaustion and recovery. The results are presented in Table 1. Our ANOVA analysis showed that there were differences in the levels of 12,13‐EpOME. In contrast, we did not detect changes in other CYP metabolites, such as 9,10‐EpOME, epoxyeicosatrienoic acids (5,6‐EET, 8,9‐EET, 11,12‐EET, 14,15‐EET), epoxyeicosatetraenoic acids (5,6‐EEQ, 8,9‐EEQ, 11,12‐EEQ, 14,15‐EEQ) and epoxydocosapentaenoic acids (7,8‐EDP, 10,11‐EDP, 13,14‐EDP, 16,17‐EDP, 19,20‐EDP) (Table 1). There were also no changes in the LOX metabolites hydroxyeicosapentaenoic acids (5‐HEPE, 8‐HEPE, 9‐HEPE, 12‐HEPE, 15‐HEPE, 18‐HEPE), hydroxyeicosatetraenoic acids (5‐HETE, 8‐HETE, 9‐HETE, 11‐HETE, 12‐HETE, 15‐HETE), hydroxydocosahexaenoic acids (4‐HDHA, 7‐HDHA, 8‐HDHA, 10‐HDHA, 11‐HDHA, 13‐HDHA, 14‐HDHA, 16‐HDHA, 17‐HDHA, 20‐HDHA, 21‐HDHA) and 13‐hydroxyoctadecadienoic acid (13‐HODE) (Table 1).

**Table 1:** Circulating plasma epoxides and diols in response to exhaustive exercise (*n* = 6)

Epoxide or diol (*μ*g/mL)	Time point 1 (rest), *P1*	Time point 2 (HF 150), *P2*	Time point 3 (exhaustion), *P3*	Time point 4 (recovery), *P4*	Greenhouse–Geisser, *P* value
9,10‐EpOME	23.45 ± 4.65	25.09 ± 7.06	28.37 ± 7.16	23.60 ± 4.87	0.235
12,13‐EpOME	31.90 ± 10.35	35.04 ± 9.16	42.91 ± 12.12	38.97 ± 13.73	Friedman *P* = 0.029
9,10‐DiHOME	7.16 ± 1.52	7.38 ± 1.58	7.24 ± 1.26	6.51 ± 0.89	0.379
12,13‐DiHOME	11.06 ± 2.61	11.63 ± 2.84	13.64 ± 4.01	13.00 ± 3.20	0.138
5,6‐EET	2.89 ± 0.56	3.29 ± 1.33	3.19 ± 1.22	2.72 ± 0.49	Friedman *P* = 0.512
8,9‐EET	4.33 ± 0.95	4.88 ± 1.91	4.53 ± 1.75	4.00 ± 0.90	0.348
11,12‐EET	2.58 ± 0.71	2.92 ± 1.04	2.72 ± 0.97	2.38 ± 0.49	0.384
14,15‐EET	3.11 ± 0.94	3.38 ± 1.06	3.11 ± 0.98	3.00 ± 0.76	0.579
5,6‐DHET	3.10 ± 0.81	3.12 ± 0.79	3.46 ± 0.92	3.16 ± 0.91	0.009
8,9‐DHET	1.83 ± 0.48	1.88 ± 0.42	2.04 ± 0.52	1.93 ± 0.58	Friedman *P* = 0.108
11,12‐DHET	0.55 ± 0.08	0.56 ± 0.05	0.59 ± 0.07	0.53 ± 0.06	0.179
14,15‐DHET	0.42 ± 0.06	0.44 ± 0.04	0.48 ± 0.05	0.45 ± 0.05	0.056
5,6‐EEQ	0.12 ± 0.04	0.12 ± 0.04	0.12 ± 0.03	0.11 ± 0.03	0.766
8,9‐EEQ	0.40 ± 0.09	0.36 ± 0.15	0.35 ± 0.11	0.33 ± 0.09	0.536
11,12‐EEQ	0.21 ± 0.08	0.21 ± 0.07	0.19 ± 0.02	0.20 ± 0.03	0.459
14,15‐EEQ	0.29 ± 0.10	0.29 ± 0.10	0.28 ± 0.08	0.25 ± 0.08	0.621
17,18‐EEQ	0.49 ± 0.16	0.49 ± 0.17	0.44 ± 0.07	0.45 ± 0.09	0.567
5,6‐DiHETE	0.99 ± 0.42	1.05 ± 0.52	1.22 ± 0.43	1.08 ± 0.46	0.019
8,9‐DiHETE	0.52 ± 0.18	0.51 ± 0.17	0.56 ± 0.19	0.52 ± 0.12	Friedman *P* = 0.163
14,15‐DiHETE	0.26 ± 0.04	0.29 ± 0.08	0.29 ± 0.06	0.23 ± 0.06	0.056
17,18‐DiHETE	1.07 ± 0.13	1.02 ± 0.25	1.29 ± 0.23	1.15 ± 0.23	0.029
7,8‐EDP	1.65 ± 0.30	1.84 ± 0.78	1.78 ± 0.75	1.53 ± 0.34	0.402
10,11‐EDP	1.50 ± 0.26	1.70 ± 0.85	1.57 ± 0.80	1.32 ± 0.36	Friedman *P* = 0.541
13,14‐EDP	0.92 ± 0.15	1.10 ± 0.40	1.03 ± 0.37	0.87 ± 0.11	Friedman *P* = 0.668
16,17‐EDP	1.18 ± 0.18	1.29 ± 0.37	1.26 ± 0.35	1.14 ± 0.15	Friedman *P* = 0.668
19,20‐EDP	1.43 ± 0.38	1.56 ± 0.47	1.61 ± 0.50	1.57 ± 0.64	Friedman *P* = 0.512
7,8‐DiHDPA	0.41 ± 0.14	0.41 ± 0.14	0.49 ± 0.17	0.45 ± 0.11	0.187
10,11‐DiHDPA	0.07 ± 0.03	0.08 ± 0.03	0.08 ± 0.03	0.07 ± 0.02	0.182
13,14‐DiHDPA	0.05 ± 0.01	0.05 ± 0.01	0.06 ± 0.01	0.05 ± 0.01	0.150
16,17‐DiHDPA	0.23 ± 0.05	0.24 ± 0.06	0.30 ± 0.10	0.26 ± 0.08	Friedman *P* = 0.056
19,20‐DiHDPA	0.20 ± 0.06	0.20 ± 0.05	0.22 ± 0.07	0.20 ± 0.06	0.249
5‐HETE	19.36 ± 3.59	21.31 ± 6.43	21.57 ± 6.04	19.39 ± 4.46	0.474
8‐HETE	8.88 ± 1.45	10.33 ± 3.80	10.13 ± 3.35	8.84 ± 1.94	0.451
9‐HETE	12.84 ± 2.85	14.97 ± 6.34	14.15 ± 5.36	12.39 ± 2.70	0.485
11‐HETE	19.86 ± 4.24	24.48 ± 10.32	22.67 ± 7.81	18.90 ± 2.94	0.358
12‐HETE	19.38 ± 4.83	20.32 ± 6.12	24.16 ± 8.19	27.09 ± 13.27	0.151
15‐HETE	10.35 ± 2.43	12.15 ± 6.40	11.54 ± 5.67	9.49 ± 2.34	Friedman *P* = 0.574
20‐HETE	0.22 ± 0.09	0.21 ± 0.09	0.22 ± 0.11	0.20 ± 0.05	0.776
5‐HEPE	2.39 ± 0.93	2.43 ± 1.12	2.30 ± 0.68	2.20 ± 0.81	Friedman *P* = 0.772
8‐HEPE	0.71 ± 0.29	0.80 ± 0.37	0.76 ± 0.19	0.71 ± 0.33	0.650
9‐HEPE	1.13 ± 0.38	1.23 ± 0.56	1.10 ± 0.37	1.07 ± 0.38	0.683
12‐HEPE	1.40 ± 0.43	1.37 ± 0.52	1.47 ± 0.48	1.58 ± 0.61	0.438
15‐HEPE	0.79 ± 0.29	0.76 ± 0.23	0.75 ± 0.21	0.71 ± 0.20	0.689
18‐HEPE	3.25 ± 0.99	3.51 ± 1.40	3.17 ± 1.08	2.86 ± 0.92	0.393
4‐HDHA	4.99 ± 1.10	6.19 ± 2.92	5.74 ± 2.80	4.68 ± .14	Friedman *P* = 0.317
7‐HDHA	3.15 ± 0.94	3.43 ± 1.45	3.54 ± 1.33	2.99 ± 0.95	0.478
8‐HDHA	6.17 ± 1.75	6.77 ± 3.18	6.41 ± 2.34	5.55 ± 1.40	0.492
10‐HDHA	3.71 ± 0.92	4.00 ± 1.57	3.93 ± 1.36	3.62 ± 0.66	0.715
11‐HDHA	3.88 ± 1.04	4.23 ± 1.85	3.97 ± 1.38	3.63 ± 0.91	0.577
13‐HDHA	3.23 ± 0.84	3.71 ± 1.82	3.45 ± 1.60	3.02 ± 0.68	0.526
14‐HDHA	10.95 ± 3.13	10.35 ± 2.75	12.05 ± 2.91	14.00 ± 5.70	Friedman *P* = 0.108
16‐HDHA	2.61 ± 0.63	3.05 ± 1.61	2.82 ± 1.41	2.28 ± 0.55	Friedman 0.073
17‐HDHA	2.97 ± 1.15	4.22 ± 1.30	4.45 ± 1.20	3.69 ± 0.90	0.097
20‐HDHA	8.97 ± 2.34	9.72 ± 4.36	9.68 ± 3.79	8.66 ± 1.83	0.660
21‐HDHA	0.38 ± 0.43	0.45 ± 0.50	0.50 ± 0.60	0.50 ± 0.56	Friedman *P* = 0.958
13‐HODE	483.37 ± 111.82	521.66 ± 140.02	529.33 ± 160.11	471.45 ± 115.38	Friedman *P* = 0.668

### Their diols

As shown in Figure 1, the main pathway of EET, EpOME, EEQ, and EDP metabolism in many cells and its conversion to DHETs, DiHOMEs, dihydroxyeicosatetraenoic acids (DiHETEs) and dihydroxydocosapentaenoic acids (DiHDPAs) by the soluble epoxide hydrolase enzyme (sEH) (Spector et al. 2004). Since acute exercise might have caused EET, EpOME, EEQ, and EDP production rapidly degraded to their diols, we analyzed the individual levels (Table 1) and the sums of the individual EETs, EpOMEs, EEQs, and EDPs and their respective diols (Table 2). We found that exercise changed 5,6‐DHET, 5,6‐DiHETE and 17,18‐DiHETE levels (Table 1). In contrast, exhaustive exercise did not change the levels of 8,9‐DHET, 14,15‐DHETE, 9,10‐DiHOME, 12,13‐DiHOME, 5,6‐DiHETE, 8,9‐DiHETE, 14,15‐DiHETE, 17,18‐DiHETE, 7,8‐DiHDPA, 10,11‐ DiHDPA, 13,14‐ DiHDPA, 16,17‐ DiHDPA, and 19,20‐ DiHDPA (Table 1). We found that exercise increased the levels of 12,13‐EpOME plus 12,13‐DiHOME (12,13‐EpOME/DiHOME), 5,6‐EET plus 5,6‐DHET (5,6‐EET/DHET), 8,9‐EET plus 8,9‐DHET (8,9‐EET/DHET), and 5,6‐EEQ plus 5,6‐DiHETE (5,6‐EEQ/DiHETE) levels, but not of the majority of metabolites (Table 2).

**Table 2:** Concentrations of individual epoxides plus their respective diols in response to exhaustive exercise (*n* = 6)

Epoxides or Diols (*μ*g/mL)	Time point 1 (rest), *P1*	Time point 2 (HF 150), *P2*	Time point 3 (exhaustion), *P3*	Time point 4 (recovery), *P4*	Greenhouse–Geisser, *P* value
9,10‐EpOME + 9,10‐DiHOME	30.20 ± 5.73	33.28 ± 8.39	35.60 ± 8.17	30.11 ± 5.41	0.263
12,13‐EpOME + 12,13‐DiHOME	42.95 ± 12.17	46.67 ± 10.87	56.56 ± 15.32	51.98 ± 16.48	0.037
5,6‐EET + 5,6‐DHET	5.98 ± 1.02	6.41 ± 2.08	6.65 ± 2.03	5.88 ± 1.26	0.037
8,9‐EET + 8,9‐DHET	6.16 ± 1.10	6.77 ± 2.27	6.57 ± 2.23	5.92 ± 1.27	0.021
11,12 EET + 11,12‐DHET	3.12 ± 0.68	3.48 ± 1.05	3.31 ± 1.00	2.90 ± 0.49	0.343
14,15‐EET + 14,15‐DHET	3.53 ± 0.95	3.83 ± 1.07	3.59 ± 0.99	3.45 ± 0.79	0.583
5,6‐EEQ + 5,6‐DiHETE	1.12 ± 0.43	1.17 ± 0.56	1.34 ± 0.45	1.19 ± 0.48	0.031
8,9‐EEQ + 8,9‐DiHETE	0.92 ± 0.22	0.87 ± 0.28	0.91 ± 0.27	0.79 ± 0.15	0.260
14,15‐EEQ + 14,15‐DiHETE	0.55 ± 0.96	0.58 ± 0.15	0.57 ± 0.10	0.48 ± 0.12	0.100
17,18‐EEQ + 17,18‐DiHETE	1.56 ± 0.14	1.51 ± 0.24	1.73 ± 0.24	1.59 ± 0.28	0.127
7,8‐EDP + 7,8‐DiHDPA	2.06 ± 0.41	2.25 ± 0.87	2.27 ± 0.89	1.98 ± 0.45	0.413
10,11‐EDP + 10,11‐DiHDPA	1.57 ± 0.28	1.78 ± 0.87	1.65 ± 0.82	1.38 ± 0.38	0.380
13,14‐EDP + 13,14‐DiHDPA	0.97 ± 0.15	1.15 ± 0.39	1.08 ± 0.37	0.92 ± 0.11	0.369
16,17‐EDP + 16,17‐DiHDPA	1.41 ± 0.18	1.53 ± 0.36	1.56 ± 0.36	1.40 ± 0.21	Friedman 0.772
19,20‐EDP + 19,20‐DiHDPA	1.64 ± 0.41	1.77 ± 0.49	1.83 ± 0.54	1.77 ± 0.70	0.415

A previous study found that submaximal testing on a bicycle ergometer caused an ~11% increase in plasma levels of 14,15‐DHET in circulating venous blood without affecting levels of 8,9‐DHET, 11,12‐DHET and respective EETs (Giordano et al. 2011). Using the maximal Bruce treadmill test, we found a similar (12%) increase in 5,6‐DHET (from 3.10 ± 0.81 to 3.46 ± 0.92 *μ*g/mL, *P* < 0.05, *t*‐test), but not in other DHETs, in Table 1. In addition, we found that exhaustive exercise increased 12,13‐EpOME levels by ~35% (from 31.90 ± 10.35 to 42.91 ± 12.12 *μ*g/mL, *P* < 0.05, *t*‐test) and 5,6‐DiHETE and 17,18‐DiHETE by ~25% each (from 0.99 ± 0.42 to 1.22 ± 0.43 *μ*g/mL and from 1.07 ± 0.13 to 1.29 ± 0.23 *μ*g/mL, respectively, for all *P* < 0.05, *t*‐tests).

### Diol/epoxide ratios

To provide insights into possible mechanisms underlying the increase in individual CYP metabolites in exercise, we calculated diol/epoxide ratios in the circulation and analyzed their changes in response to exhaustive exercise and post exercise. The results are presented in Table 3. Our ANOVA analysis showed that exercise did not change the ratios for each substrate class in vivo during and post exercise. Similar results were found for the individual metabolites, as shown in Table 4. We also found that the four classes of epoxy‐metabolites are unequally hydrolyzed to appear in the circulation (Greenhouse‐Geisser, *P1*,* P* = 0.001). We found that EEQs are preferentially metabolized into their diols (ratio DiHETEs/EEQs at *P1*, 1.97 ± 0.61) compared to EpOMEs, EETs and EDPs (ratios diols/epoxy‐metabolites at *P1*, 0.34 ± 0.05, 0.47 ± 0.15, 0.15 ± 0.04, respectively) in Table 2. In fact, the following order of ratios was identified: DiHETEs/EEQs > DHETs/EETs = DiHOMEs/EpOMEs > DiHDPA/EDPs (paired *t*‐tests, one‐tailed, Bonferroni correction). This pattern was also found for individual metabolites, as shown in Table 4. Together the results indicate that 5,6‐DHET, 9,10‐EpOME, 5,6‐DiHETE, and 17,18‐DiHETE are rather released and accumulated in the circulation during the hemodynamic and metabolic response in short‐term maximal exercise than result from altered sEH activity.

**Table 3:** Ratios estimated using total concentrations of epoxides and diols in response to exhaustive exercise (*n* = 6)

Epoxides or diols (*μ*g/mL) or Ratios	Time point 1 (rest), *P1*	Time point 2 (HF 150), *P2*	Time point 3 (exhaustion), *P3*	Time point 4 (recovery), *P4*	Greenhouse–Geisser, *P* value
9,10‐EpOME + 12,13‐EpOME	54.94 ± 14.25	60.94 ± 14.14	71.28 ± 16.82	62.57 ± 16.78	0.093
9,10‐DiHOME + 12,13‐DiHOME	18.22 ± 3.85	19.01 ± 4.21	20.88 ± 4.98	19.52 ± 3.66	0.383
Ratio (9,10‐DiHOME + 12,13‐DiHOME)/(9,10‐EpOME + 12,13‐EpOME)	0.34 ± 0.05	0.32 ± 0.06	0.30 ± 0.05	0.31 ± 0.05	0.189
5,6‐EET + 8,9‐EET + 11,12 EET + 14,15‐EET	12.90 ± 3.11	14.47 ± 5.24	13.56 ± 4.85	12.10 ± 2.47	0.407
5,6‐DHET + 8,9‐DHET + 11,12‐DHET ± 14,15‐DHET	5.90 ± 1.35	6.01 ± 1.21	6.58 ± 1.48	6.06 ± 1.51	0.031
Ratio (5,6‐DHET + 8,9‐DHET + 11,12‐DHET + 14,15‐DHET)/(5,6‐EET + 8,9‐EET + 11,12 EET + 14,15‐EET)	0.47 ± 0.15	0.44 ± 0.10	0.51 ± 0.10	0.51 ± 0.12	0.461
5,6‐EEQ + 8,9‐EEQ + 11,12‐EEQ + 14,15‐EEQ + 17,18‐EEQ	1.52 ± 0.43	1.49 ± 0.52	1.39 ± 0.28	1.35 ± 0.30	0.540
5,6‐DiHETE + 8,9‐DiHETE + 14,15‐DiHETE + 17,18‐DiHETE	2.84 ± 0.65	2.88 ± 0.80	3.37 ± 0.67	2.92 ± 0.63	0.018
Ratio (5,6‐DiHETE + 8,9‐DiHETE + 14,15‐DiHETE + 17,18‐DiHETE)/(5,6‐EEQ + 8,9‐EEQ + 11,12‐EEQ + 14,15‐EEQ + 17,18‐EEQ)	1.97 ± 0.61	2.08 ± 0.81	2.46 ± 0.50	2.21 ± 0.48	0.237
7,8‐EDP + 10,11‐EDP + 13,14‐EDP + 16,17‐EDP + 19,20‐EDP	6.68 ± 0.83	7.50 ± 2.38	7.25 ± 2.30	6.43 ± 0.87	0.450
7,8‐DiHDPA + 10,11‐DiHDPA + 13,14‐DiHDPA + 16,17‐DiHDPA + 19,20‐DiHDPA	0.97 ± 0.25	0.99 ± 0.22	1.15 ± 0.31	1.04 ± 0.23	0.042
Ratio (7,8‐DiHDPA + 10,11‐DiHDPA + 13,14‐DiHDPA + 16,17‐DiHDPA + 19,20‐DiHDPA)/(7,8‐EDP + 10,11‐EDP + 13,14‐EDP + 16,17‐EDP + 19,20‐EDP)	0.15 ± 0.04	0.14 ± 0.04	0.16 ± 0.04	0.16 ± 0.02	0.357

**Table 4:** Ratios estimated using individual concentrations of epoxides and their diols in response to exhaustive exercise (*n* = 6)

Ratios	Time point 1 (rest), *P1*	Time point 2 (HF 150), *P2*	Time point 3 (exhaustion), *P3*	Time point 4 (recovery), *P4*	Greenhouse–Geisser, *P* value
9,10‐DiHOME/9,10‐EpOME	0.314 ± 0.054	0.292 ± 0.046	0.260 ± 0.038	0.281 ± 0.040	0.169
12,13‐DiHOME/12,13‐EpOME	0.357 ± 0.070	0.341 ± 0.076	0.323 ± 0.062	0.347 ± 0.067	0.279
5,6‐DHET/5,6‐EET	1.100 ± 0.330	0.994 ± 0.172	1.135 ± 0.246	1.160 ± 0.257	0.444
8,9‐DHET/8,9‐EET	0.439 ± 0.136	0.413 ± 0.110	0.475 ± 0.115	0.491 ± 0.121	0.388
11,12‐DHET/11,12‐EET	0.228 ± 0.080	0.213 ± 0.079	0.241 ± 0.083	0.229 ± 0.046	0.749
14,15‐DHET/14,15‐EET	0.147 ± 0.046	0.144 ± 0.050	0.168 ± 0.048	0.154 ± 0.024	0.401
5,6‐DiHETE/5,6‐EEQ	8.617 ± 3.927	8.512 ± 2.270	10.093 ± 2.401	9.698 ± 2.820	0.370
8,9‐DiHETE/8,9‐EEQ	1.318 ± 0.499	1.551 ± 0.591	1.643 ± 0.549	1.449 ± 0.510	0.439
14,15‐DiHETE/14,15‐EEQ	0.977 ± 0.418	1.103 ± 0.505	1.152 ± 0.520	0.957 ± 0.349	0.512
17,18‐DiHETE/17,18‐EEQ	2.441 ± 0.954	2.428 ± 1.401	2.972 ± 0.739	2.624 ± 0.575	0.380
7,8‐DiHDPA/7,8‐EDP	0.247 ± 0.072	0.234 ± 0.060	0.287 ± 0.076	0.295 ± 0.018	0.226
10,11‐DiHDPA/10,11‐EDP	0.049 ± 0.013	0.048 ± 0.013	0.055 ± 0.016	0.055 ± 0.008	0.582
13,14‐DiHDPA/13,14‐EDP	0.055 ± 0.017	0.050 ± 0.015	0.058 ± 0.014	0.057 ± 0.005	0.631
16,17‐DiHDPA/16,17‐EDP	0.203 ± 0.060	0.201 ± 0.072	0.248 ± 0.090	0.226 ± 0.045	0.352
19,20‐DiHDPA/19,20‐EDP	0.146 ± 0.039	0.134 ± 0.037	0.144 ± 0.038	0.132 ± 0.021	0.545

## Discussion

This study is the first documentation that circulating endogenous *n* − 3 and *n* − 6 CYP mediator levels can be modulated by short‐term exhaustive exercise in humans. In particular, we found that the circulating levels of the CYP metabolites 12,13‐EpOME/DiHOME, 5,6‐EET/DHET, 5,6‐EEQ/DiHETE, and 17,18‐EEQ/DiHETE increased in response to maximal exercise. We found that these changes are unlikely related to altered sEH activity, which preferentially metabolizes other CYP expoxy‐metabolites (i.e., EEQs) into their diols (Fischer et al. 2014). We did not detect changes in specific mediators derived from the LOX and CYP omega/(omega‐1) pathways in response to exhaustive exercise. The extent to which beneficial cardiovascular effects of *n* − 3 polyunsaturated fatty acids (PUFAs) and cardiovascular exercise are mediated by increased levels of epoxides derived from the CYP monooxygenase pathway remains to be explored.

### EETs/DHETs

Endothelial cells are reservoirs of EETs and the primary source of plasma EETs, which are esterified to the phospholipids of lipoproteins (Jiang et al. 2010; Jiang et al. 2011; Schunck et al. 2017). On release, EETs affect vascular tone and blood pressure, produce pro‐fibrinolysis and reduce inflammation (Jiang et al. 2010; Jiang et al. 2011; Jiang et al. 2012). DHETs were initially thought to be inactivation products of EETs, but several recent studies indicate that, like EETs, they produce vasodilation (Hercule et al. 2009) and activate smooth muscle BK channels (Lu et al. 2001). The mechanisms of how epoxides and diols are released from the tissues and eventually become constituents of circulating lipoproteins are largely unknown, making it difficult to explain these findings. Cells preferentially release DHETs while storing the EETs (Roman 2002), suggesting that certain diols might be overrepresented in the circulation compared with the epoxide/diol ratios in the tissues where these metabolites were primarily produced (Fischer et al. 2014). In this study, we were able to demonstrate that maximal treadmill exercise affects plasma EET/DHET status, in particular by increasing 5,6‐DHET levels, which could contribute to cardiovascular responses and hemodynamics. Of note, submaximal testing on a bicycle ergometer has been found to increase plasma levels of 14,15‐DHET (Giordano et al. 2011). Our data are in agreement with the idea that EETs/DHETs are attractive mediator signals for skeletal muscle blood flow regulation in humans because they can act as potent vasodilators (Campbell and Fleming 2010) and their circulating levels are increased during exercise. These properties could be essential to counteract the vasoconstrictor effects of concurrent increases in muscle sympathetic nerve activity and circulating vasoconstrictor substances during exercise.

### 12,13‐EpOME

We observed increases in plasma concentrations of 12,13‐EpOME during maximal exercise. However, 9,10‐EpOME and 12,13‐EpOME (leukotoxins A and B) were initially described to be produced by neutrophils during the oxidative burst to combat bacterial infection (Thompson and Hammock 2007). These mediators are converted by sEH into DiHOMEs (Fig. 1), which suppress the neutrophil respiratory burst (Thompson and Hammock 2007). Recent findings suggest that both EpOMEs (with 12,13‐EpOME being more potent) can exhibit both cardiodepressant (Sugiyama et al. 1987; Fukushima et al. 1988; Siegfried et al. 1990) and vasoactive properties, for example, by increased endothelial NO and O(2)(*‐) production (Okamura et al. 2002). Increased cardiac tissue levels of DiHOME may cause detrimental effects on postischemic cardiac function (Chaudhary et al. 2013; Bannehr et al. 2019). Therefore, it is unclear whether the observed increases in plasma levels of 12,13‐EpOME in our study reflect a physiological cardiovascular response upon exercise or may potentially contribute to detrimental cardiovascular effects. Interestingly, elite and nonelite athletes have an increased incidence of sudden death (Del Rio‐Santiago et al. 2012; Bohm et al. 2016), which has been attributed to a low omega‐3 index, that is, low red blood cell EPA/DHA status (von Schacky et al. 2014). Our study involved nontrained subjects. It is unknown whether increases in plasma levels of 12,13‐EpOME are also observed in trained athletes and in extreme endurance training. Nevertheless, our study indicates that circulating CYP products of LA, that is, EpOMEs, deserve special attention in extreme exercise. Our data also indicate that these metabolites could also affect hemodynamics in these conditions. Of note, we found that the majority of CYP and LOX metabolites measured were not affected, suggesting that these metabolites are unlikely to play a role in this scenario.

### 5,6‐DiHETE and 17,18‐DiHETE

We observed increases in plasma concentrations of 5,6‐DiHETE and 17,18‐DiHETE during maximal exercise. These mediators are produced by hydrolysis of 5,6‐EEQ and 17,18‐EEQ, respectively (Fig. 1). While the putative biological function(s) of 5,6‐DiHETE and 17,18‐DiHETE have not received much attention, 17,18‐EEQ has been identified as potent vascular BK_Ca_ channel activator and vasodilator, which is more potent that EETs (Lauterbach et al. 2002; Hercule et al. 2007). Inhibition of sEH is a potential approach for enhancing the biological activity of EETs (Spector and Kim 2015). However, presumably higher levels of EETs in blood and tissue in vivo may have also detrimental cardiovascular side effects (Gschwendtner et al. 2008; Hutchens et al. 2008; Wutzler et al. 2013). We have no evidence that the higher levels of 5,6‐DHET, 5,6‐DiHETE, and 17,18‐DiHETE during maximal exercise result from sEH enzyme activation. Based on our calculations of diol/epoxide ratios, this is rather unlikely. Furthermore, the levels of other DHETs and DiHETEs, DiHOMEs, and DiHDPAs did not vary during maximal exercise. The mechanisms of how epoxides and diols are released from the tissues and eventually become constituents of circulating lipoproteins are largely unknown, making it difficult to explain these findings (Fischer et al. 2014). Nevertheless, the function and pathophysiological roles of circulating 5,6‐DiHETE and 17,18‐DiHETE has yet to be integrated into a physiological and pathophysiological context. This is particularly important since drugs that mimic 17,18‐EEQ have been developed and are viewed as novel drug candidates that may overcome limitations of dietary EPA/DHA supplementation for cardiovascular health benefits (Schunck et al. 2017).

### Exercise protocol considerations and limitations

The modified Bruce cardiac stress test was used to ensure that all runners were able to complete a similar highest intensity workload (13.50 ± 1.97 METs) concomitant with robust and significant short‐term increases in hemodynamics (heart rate and blood pressure) without fatiguing. For consistency, we also used an intermediate workload (10.00 ± 1.90 METs, *P* = 0.0009, *t*‐test) reaching a heart frequency of 150 or more beats per minute to ensure that all subjects could complete the test with profound increases in heart rate and blood pressure without fatiguing. Our clinical data (Fig. 3) show that the exercise protocol caused the expected hemodynamic changes in our study.

We obtained venous blood from an arm vein, although the source of plasma lipid mediators, if modified from exercising muscle and its vasculature, suggests that the leg would provide a different measure. However, blood samples were taken from the arm vein because of the great difficulty associated with obtaining blood from a vein in dynamically contracting leg muscles. Therefore, stronger effects may have been present in the venous effluent of the exercising muscle (Giordano et al. 2011). We studied effects of maximal short‐term exercise, but not endurance exercise, which may also lead to different results.

## Conclusions

To the best of our knowledge, we conducted the first study on the impact of acute exercise on individual circulating fatty acid epoxy‐metabolites derived from the CYP monooxygenase pathway and specific mediators derived from the LOX and CYP hydrolase pathways. Lipid mediator profiling was performed on venous blood taken from healthy individuals undergoing maximal treadmill exercise using the standard Bruce protocol to induce strong and robust hemodynamic and metabolic changes (Bruce et al. 1973; Trappe and Lollgen 2000). We confirmed our hypothesis that individual epoxy‐metabolites are modulated in response to short‐term exhaustive exercise (with exception of EEQ and LOX metabolites). The changes concurred with profound effects on heart rate, blood pressure, and lactate. Our results indicate that individual circulating CYP epoxy‐metabolite status, particularly 12,13‐EpOME/DiHOME, 5,6‐EET/DHET, 5,6‐EEQ/DiHETE, and 17,18‐EEQ/DiHETE status, may affect the cardiovascular response in short‐term maximal exercise. Future research is required to determine the contribution of the individual epoxy‐metabolites to cardiac performance and regulation of coronary and/or skeletal‐muscle blood flow in health and cardiovascular disease.

## Conflict of Interest

None.

## Supporting information




**Table S1.** Bruce protocol and estimated metabolic equivalents of task (METs).
