# Supplementary figures and images for: Maximal exercise and plasma cytochrome P450 and lipoxygenase mediators: a lipidomics study
Source: Physiol Rep. 2019 Jul 15;7(13):e14165. doi: 10.14814/phy2.14165 (PMC6640589; doi:10.14814/phy2.14165)

## Slide 1
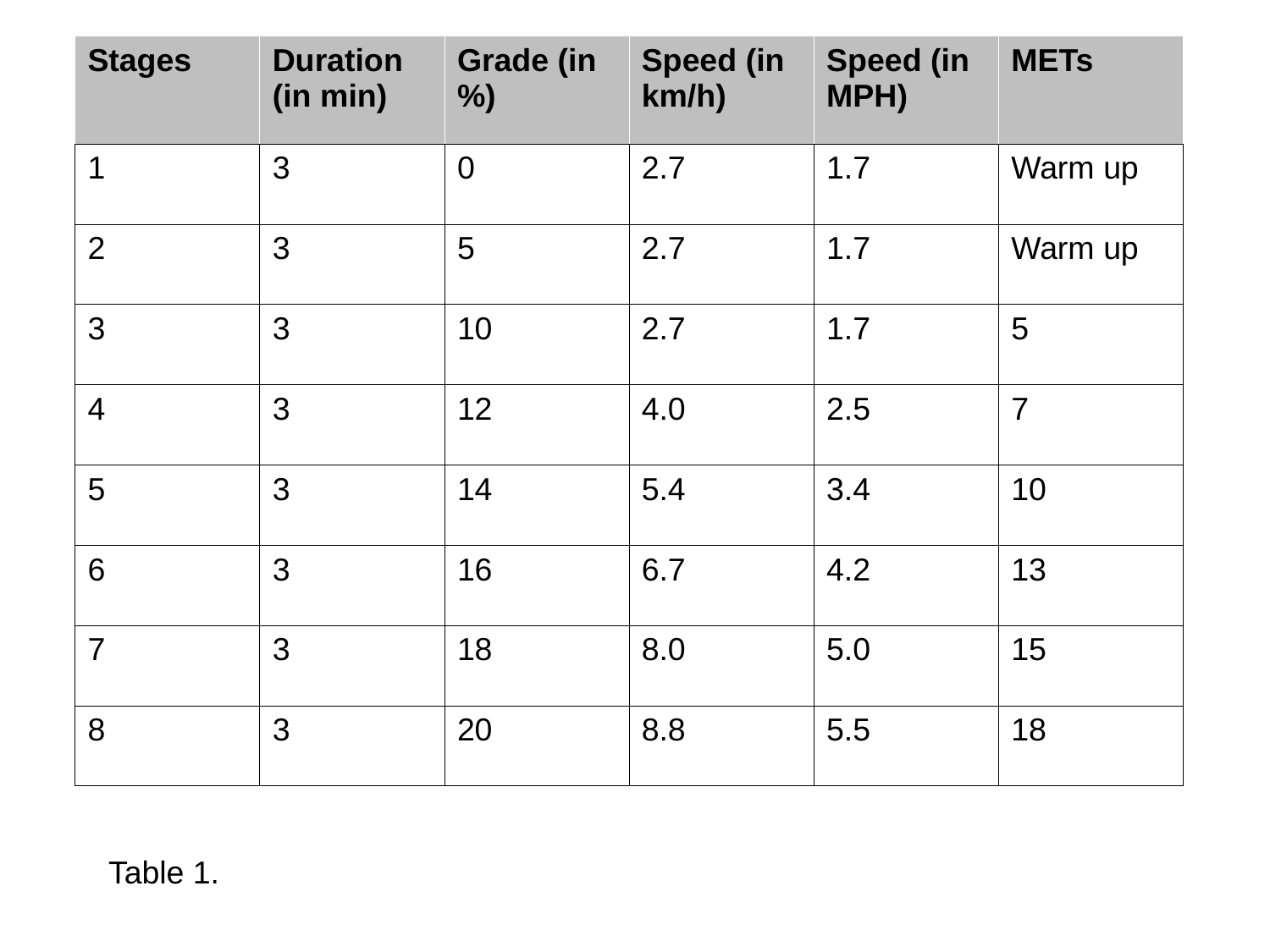

| Stages | Duration (in min) | Grade (in %) | Speed (in km/h) | Speed (in MPH) | METs |
| --- | --- | --- | --- | --- | --- |
| 1 | 3 | 0 | 2.7 | 1.7 | Warm up |
| 2 | 3 | 5 | 2.7 | 1.7 | Warm up |
| 3 | 3 | 10 | 2.7 | 1.7 | 5 |
| 4 | 3 | 12 | 4.0 | 2.5 | 7 |
| 5 | 3 | 14 | 5.4 | 3.4 | 10 |
| 6 | 3 | 16 | 6.7 | 4.2 | 13 |
| 7 | 3 | 18 | 8.0 | 5.0 | 15 |
| 8 | 3 | 20 | 8.8 | 5.5 | 18 |
Table 1.

Supplement: Supplementary file 1 — Table S1. Bruce protocol and estimated metabolic equivalents of task (METs). [file PHY2-7-e14165-s001.ppt]
