# Supplementary material for: Maximal exercise and plasma cytochrome P450 and lipoxygenase mediators: a lipidomics study
Source: Physiol Rep. 2019 Jul 15;7(13):e14165. doi: 10.14814/phy2.14165 (PMC6640589; doi:10.14814/phy2.14165)
Supplement: Supplementary file 2 [file PHY2-7-e14165-s002.docx]

**Abbreviations**

PUFA, polyunsaturated fatty acid;

CYP, cytochrome P450;

LOX, lipoxygenase;

HODE , hydroxyoctadecadienoic acid;

HpODE, hydroperoxylinoleic acid

EpOME, epoxyoctadecenoic acid;

DiHOME, dihydroxyctadecenoic acid;

EET, epoxyeicosatrienoic acid;

EEQ, epoxyeicosatetraenoic acid;

EDP, epoxydocosapentaenoic acid;

DHET, dihydroxyeicosatrienoic acid;

DiHETE, dihydroxyeicosatetraenoic acid;

DiHDPA, dihydroxydocosapentaenoic acid;

HETE, hydroxyeicosatetraenoic acid;

HPETE, hydroperoxyeicosatetraenoic acid;

HEPE, hydroxyeicosapentaenoic acid;

HDHA, hydroxydocosahexaenoic acid;

LTB, leukotriene B;

LXA, lipoxin A;

DiHDHA, dihydroxydocosahexaenoic acid;

LA, linoleic acid, C18:2;

EPA, eicosapentaenoic acid, C20:5 n-3;

AA, arachidonic acid, C20:4;

DHA, docosahexaenoic acid,C22:6 n-3;

EDHF, endothelium-derived hyperpolarizing factor;

ATP, adenosine 5’-triphosphate;

**Author contributions**

BG and FCL planned and designed the experimental studies. BG conducted and guided the treadmill experiments. ID and MR performed the LC–MS/MS spectrometry experiments. All authors contributed to the implementation and analyses of the experiments. BG and FCL drafted the article, and all authors, contributed to its completion.
